# Epigenetic–Genetic Coupling and Understanding the Molecular and Cellular Basis of Lamarckian Inheritance

**DOI:** 10.3390/ijms27042003

**Published:** 2026-02-20

**Authors:** Robyn A. Lindley, Reginald M. Gorczynski, Edward J. Steele

**Affiliations:** 1 Department Clinical Pathology, Victorian Comprehensive Cancer Centre (VCCC), University of Melbourne, Melbourne 3052, Australia; robyn.lindley@unimelb.edu.au; 2 GMDx Group Ltd., 117 Hawthorn East, Melbourne 3123, Australia; 3 Institute of Medical Sciences, University of Toronto, Toronto, ON M5G 1X5, Canada; reggorczynski@gmail.com; 4 Melville Analytics Pty Ltd. and Immunomics, Brisbane 4169, Australia

**Keywords:** epigenetic inheritance, hard lamarckian inheritance, deaminase driven reverse transcription mutagenesis (DRT), target site reverse transcription (TSRT), DNA polymerase eta, soma to germline feedback, immunoglobulin somatic hypermutation, single molecular real time (SMRT) genomic sequencing, IGHV locus in humans and mice

## Abstract

This critical and selective review synthesizes the accumulating body of biological evidence supporting a process we term *epigenetic–genetic coupling* as a mechanistic basis for Lamarckian inheritance of somatically acquired adaptations. We propose that evolutionary processes in mammals and higher vertebrates can involve deaminase-driven, reverse transcriptase-mediated, RNA-templated targeted homologous recombination. We contrast well-established examples of “Soft”, reversible epigenetic inheritance with historical and contemporary evidence suggestive of stable, DNA-integrated “Hard” Lamarckian transgenerational inheritance. Our analysis indicates that the establishment of “Hard” Lamarckian inheritance may require specific population dynamics, including inbreeding or interbreeding among phenotypically affected offspring, together with sustained and defined environmental stimuli over one or more generations to consolidate the acquired traits at the genomic level. We also present molecular and cellular evidence supporting RNA-to-DNA genetic feedback mechanisms involving targeted genomic integration, primarily mediated by the DNA repair–associated reverse transcriptase activity of DNA polymerase η. Finally, we review diversification mechanisms in molecular and cellular immunology that now routinely employ single-molecule, real-time, long-read genomic sequencing (6–8 kb). We recommend the broader application of these technologies in future breeding and experimental programs across other somatic systems. Their deployment offers a robust strategy for securing definitive “Hard” molecular evidence of Lamarckian acquired inheritance in diverse biological contexts; including somatically acquired immunity, as well as adaptive behavioral and central nervous system phenotypes. This is compatible with our over-arching goal—to provide an experimental road map of conceptual options to drive future experimentation in acquired inheritance breeding programs.

## 1. Introduction—Conceptual Overview

**Figure 1:**
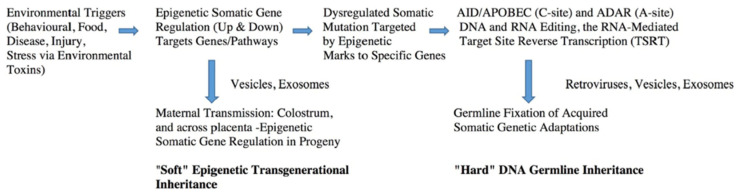
Coupled Epigenetic-Genetic Mechanisms of Lamarckian Inheritance with the Penetration of the Weismann Barrier in Higher Animals. Adapted from Steele et al. published in 2019 [10]. See Figure 2 and associated text and references from that paper.

At the start we would like to point out that our aim is to critically review the mounting body of experimentally demonstrated direct evidence allowing us to advance the concept of what we term “Epigenetic–Genetic” coupling driving Lamarckian inheritance of somatically acquired characteristics. The term “Lamarckian inheritance” is used in a mechanistic and operational sense to describe the specific molecular processes leading to acquired transgenerational observations discussed in the review. In our view all Darwinian natural selection principles still apply, except now the combined experimental data also imply that RNA-to-DNA hard inheritance underpins the process, rather than implying a departure from established evolutionary theory. To help guide the reader a schematic distinguishing the clear experimentally supported ‘Soft’ and ‘Hard’ aspects of epigenetic initiated and hard inheritance is shown in Figure 1. At the outset we also point to Section 5, Section 6 and Section 7 where we review the direct experimental evidence for the RNA-to-DNA feedback step established in somatic human cells, driven by the reverse transcriptase functions of DNA Polymerase eta (η). We hypothesize here that this provides the stable transgenerational inheritance often observed. We thus advance the hypothesis, supported by direct experimental evidence, that the evolutionary process is underpinned by the experimental and observational evidence supporting deaminase-driven reverse transcriptase (DRT)-mediated RNA-templated homologous recombination. Thus, these molecular steps are in turn a plausible hypothesized explanation for all known examples of stable transgenerational Lamarckian acquired inheritance phenomena observed in mammals and higher vertebrates.

This critical review is therefore *not about* the deeper historical narratives and old debates distinguishing Lamarckian Inheritance versus Darwinian Natural Selection that date to both Lamarck and Darwin in the 18th and 19th Centuries. From our publication history [1,2,3,4,5,6,7,8,9,10] it is clear we think they are intertwined inheritance processes. The major distinction being that Lamarckian evolution must be much faster in real time than the traditional slower expectations of Darwinian Natural Selection Theory. This is again because at the molecular level DNA Polymerase eta (η)-driven RNA-back-to-DNA is now a real experimental phenomenon in human cells (Section 7).

Since the late 1970s we have published scholarly books, and reviews, and semi-popular magazine articles discussing this deeper history and experimental observations [1,2,3,4,5,6,7,8,9,10] so the reader *should not expect* this paper to cover all that older narrative literature again, e.g., discussion on Use v Disuse of organs, Weismann Barrier, the tail chopping experiments, distortion of modern genetic science by Lysenko, Darwin’s own Lamarckian theory of ‘Pangenesis’, etc. On these issues Liu and colleagues have also recently published several perceptive historical articles [11,12,13]. Nor should the reader expect a wider disquisition on the impact of inbreeding on global genetic diversity patterns, although we deal with that tangentially in a historical sense. The ‘inbreeding concept’ is introduced here as an *experimental tool* to help distinguish in future experimentation ‘Soft’ or reversible epigenetic acquired inheritance from ‘Hard’ or DNA-stable genetic acquired inheritance. As stated in the opening paragraph our focused aim is to discuss and analyze only “experimentally demonstrated direct evidence” or hard observations from transgenerational breeding programs or natural observations in multigenerational human families we expect to see in the future.

We therefore present an up-to-date analytical review—both evidence-based, yet selective—of transgenerational acquired inheritance breeding data and observations published over the past 50 to 100 years—as well as key epigenetics and long noncoding RNA (lncRNA) papers which have been published more recently [14,15,16,17,18,19,20,21,22,23,24,25,26,27,28,29,30,31,32]. Because the topic is of fundamental interest, the paper is written in a plain-language scientific style. We are traditional immunologists, immunogeneticists and bioinformaticists. Our pertinent work on the role of the somatic hypermutation (SHM) process has been published since the late 1970s and is cited throughout the article. The SHM concepts are central to our body of work (Section 5, Section 6 and Section 7). In this way we hope to communicate with a wide scientific audience. However, there is a clear focus on recent Epigenetic breeding experiments in laboratory animals [14,15,16,17,18,19,20,21,22,23,24,25,26] and human observations, as well as detailed genetic observations and experiments on immune IgV region receptor diversity arrays published by various experimental communities in recent years [33,34,35,36,37,38,39,40,41,42,43,44,45,46,47,48,49]. The latter discussion includes a ‘tutorial’ type outline on the importance of understanding the well-studied “V to DJ” somatic rearrangement process in the immune system (Section 8). We include this because many biomedical scientists and non-immunologists have a poor understanding of what that somatic genetic rearrangement process is and then what SHM really implies for germline sequence structure [4,5]. Some terms often found difficult to understand are in the Box and expanded further in the Supplementary Material.

The paper has been structured into nine parts.

- First, we address how we may reconcile the recent solid examples of ‘Soft’ or reversible epigenetic acquired inheritance and contrast it with other historical evidence of apparently stable DNA-integrated, or what we hereafter refer to as ‘Hard’, Lamarckian transgenerational acquired inheritance (Section 2).
- Second, we analyze more critically the ‘reversible’ interpretative aspects of current epigenetic inheritance breeding experiments. We show that these fall short of an adequate explanation of what is observed, which could be stable (‘Hard’) Lamarckian inheritance (Section 3).
- We then propose that ‘Hard’ Lamarckian inheritance may require forms of inbreeding and inter-breeding among putative phenotypically affected offspring populations. We see this as coupled to sustained specific ‘environmental stimulation’ and selection acting over more than one generation to experimentally lock in a ‘Hard’ inheritance phenomenon (Section 4).
- Next, we transition to RNA back to DNA feedback loops as current known molecular mechanisms in the immune system and cancer progression at immunoglobulin (Ig) and non-Ig loci in Section 5, Section 6 and Section 7. These sections provide strong indirect evidence as well as solid hard direct experimental evidence. This supports our proposal that donor nucleic acids (mainly RNAs both short and long), following targeted genetic loci recognition, can integrate into their target DNA base sequences and replace them by an RNA-templated homologous recombination process. This step involves the ubiquitous DNA repair enzyme DNA polymerase (η) eta (viz. TSRT) both in the nuclear genome of both somatic cells and, we predict, in the germline genome of reproductive cells, ova and spermatozoa.
- Finally, in Section 8 and Section 9 we analyze at more depth known diversification mechanisms in molecular and cellular immunology. We discuss the now-routine deployment by the Watson group and the Collins group of innovative single-molecule real-time (SMRT) long-read (6–8 kb) genomic sequencing. This type of sequencing allows accurate assembly of large, highly repetitive, loci of similar sequences. This is demonstrated for the human 1 Mb IGHV haplotype region, where it can now be performed with 100% accuracy to nucleotide resolution, a feat not hitherto possible by current short-read 300 bp NGS genomic sequencing. This detailed discussion is very revealing. It can provide future breeding programs with the technical-genetic tools, and intellectual strategies, needed to secure compelling evidence demonstrating Lamarckian acquired inheritance.

At the outset we acknowledge the work of several key groups that have contributed substantially to the development of this field. They include Spadafora and his colleagues [14,15,16]; Rassoulzedegan and her colleagues [17,18]; Dias, Ressler and their colleagues [19,20]; Rando, Sharma and their colleagues [21,22]; and Mansuy and her colleagues [23,24,25,26]. We also acknowledge the conceptual coherence and experimental work of the Mattick group over many years as a major contribution to the field [27,28,29,30,31]. Lastly, we acknowledge the technical innovations and strategic systematic applications which have enhanced our understanding (to nucleotide level resolution) of the extensive inherited diversity of the Antibody and T Cell Receptor antigen recognition repertoires by the Watson [33,34,35,36,37,38,39,40,41,42,43] and Collins [44,45,46,47,48,49] groups.

## 2. Epigenetic–Genetic Coupling’ and Transition from ‘Soft’ to ‘Hard’ Lamarckian Inheritance

Seven years ago, we presented a plausible set of Lamarckian-type mechanisms and processes that we argued could facilitate the rapid genetic evolution of life, and indeed facilitate the spreading of life, throughout the cosmos [10]. In that paper we introduced the emerging concept of ‘Epigenetic–Genetic Coupling’. This is still a theoretical process which we feel helps understand the transition from ‘Soft’ to ‘Hard’ Lamarckian inheritance of acquired somatic characteristics. Our aim in what follows is to develop a plausible answer to the question: Can we, and if so, how do we, reconcile the numerous recent examples of ‘Soft’ or reversible epigenetic acquired inheritance with other historical examples of apparently stable DNA-integrated, or ‘Hard’ Lamarckian transgenerational acquired inheritance?

By soft epigenetic inheritance we refer to transgenerational phenomena including, but not limited to, ‘The Dutch Famine’ post-World War II [50]; parental olfactory experience influencing both behavior and neural structure in subsequent generations [19,20]; epigenetic inheritance of the effects of postnatal behavioral trauma [23,24,25], by inducing post-natal maternal stress and transgenerational behavioral and metabolic traits through the male line to the 5th and 6th generations [26]. Other recent clear studies relevant to COVID-19 protective mucosal immunity have involved “trained” acquired inheritance of nonspecific or heterologous innate immunity also via the male line [51,52,53].

These recent epigenetic studies involving sperm-carrying RNA regulatory molecules followed the breakthrough 2006 work in mice of Rassoulzedegan and associates [17]. They demonstrated that microinjection of total RNA into fertilized eggs from Kit tm1Alf/+ heterozygotes allowed for subsequent demonstration that Kit-specific microRNAs (miRNA) themselves transmitted a heritable white tail phenotype to progeny. They thus identified a mode of epigenetic inheritance associated with the zygotic transfer of RNA molecules. These phenomena occurred via the male line, were reversible, and while persisting over the generations to at least the 5th generation were already waning by the 6th generation as documented in a recent definitive F_1_–F_6_ study discussed further below [26].

Nevertheless, there are other hard-wired acquired inheritance observations that we have reviewed over the past 50 years that seem to “deepen in intensity” in subsequent generations [1,2,10,54,55]. The stable genetic signature of these acquired inheritance phenomena is particularly apparent in the immune system at the genomic loci encoding specific antigen binding Ig variable region molecules of the immune system. This is documented by the inferred putative somatic origins and then stable germline inheritance of diverse yet highly similar Ig variable (V) region elements in tandem in long haplotype gene arrays in mice and humans [4,5,56]. A similar stable genetic situation applies to the evolution of the tandem arrays of T Cell receptor (TCR) germline V gene families [57] which are also inherited as long genomic haplotypes. This is discussed in the context of the putative somatic hypermutation origin of novel TCRs. We speculate that their maintenance is via ‘germline tracking’ within diverse T lymphocyte expressed V gene families. We thus speculate this represents a soma-to-germline “tracking” or “monitoring” mechanism for newly emergent somatic alleles of MHC Class I and II antigenic peptide presenting molecules [57].

However, there is also much observational evidence of clear inheritance of what must have been originally acquired and specific instinctual responses in our ancestors as well as many other animal and invertebrate species in the deep evolutionary past, e.g., classic flight or fight visual fear and olfactory instincts in mammals. There are other examples of ‘Hard’ acquired inheritance phenomena in higher animals also involving the immune system as documented in the pioneering experiments of Guyer and Smith conducted in 1918–1924 (at University of Wisconsin), where there was an apparent deepening in autoimmune destructive intensity down the generations [1,2,55] (full reference details are in Steele 2016 [55] with a reprinted example from the Guyer and Smith papers). Other examples involve induced metabolic and hormonal disorders [58] and many other examples reviewed in depth in a 2010 book by one of us, Lindley 2010 [7]. The acquired diabetes studies are especially informative and are based on the experimental data published in 1972 by Goldner and Spergel who confirmed and extended the original alloxan-induced transgenerational diabetic phenomena [59] published earlier in many small laboratory animals by Okamoto [60]. Other observations published in the literature in the past 50–100 years are reviewed elsewhere [2,10] and in the selected examples discussed in some detail below (Section 4).

How can we plausibly reconcile these sets of apparently contradictory observations for long-term (through generations) Lamarckian inheritance with the more recent epigenetic paternal inheritance observations? These effects, while rapidly appearing in F_1_ and, since via the male, ‘Lamarckian-like’ are thus immediately adaptive. However, they are epigenetic and thus “above” the DNA genes and thus considered ‘Soft’ and *reversible inheritance*. More recent reviews by others have re-emphasized this apparent anomaly [61,62]. The central concepts and steps towards a plausible explanation were summarized earlier in a book by Jablonka and Lamb [63] and then later in 2010 by Lindley [7]. All these observations are summarized as a schematic in Figure 1. This schematic summary remains valid in our opinion in surveys of the current literature and serves as the focal point here for further discussion.

Note the two phases of response are via targeted elevated nuclear gene expression. While much of this locus targeting, no doubt involving specific transcription factor complexes and demethylation and chromatin acetylation events, remains obscure [10], this epigenetic “opening up” and chromatin remodeling is essential, we consider the special role for specific ‘Triplex’ dsDNA sequence recognition is important (Section 7). Note the hypothesized important roles for vesicles and exosomes as intercellular communication vehicles bearing shuttled cargoes from donor cells to target cells of protein factors, small hormonal molecules as well as small and large RNA and DNA informational molecules.

In the first, which is exclusively epigenetic, upregulation of transcriptional expression of selected suites of coordinated loci by environmental triggers (whether endogenous or external to the body) as a mature multicellular sexually differentiated animal initiates a ‘somatic genetic’ and thus ‘somatic selection’ response [1,2]. The targeted protein coding gene sets are turned on by transcription factors and other epigenetic chromatin-opening enzymes. The higher levels of transcription rate and appearance of associated gene products (and we assume pre-mRNAs, long non-coding lncRNAs, short regulatory miRNAs, etc.) can then lead to membrane-bounded vesicle/exosomal transmission to other cells of the produced protein and RNA cargos of key functional and informational molecules. Indeed, the vesicle/exosomal cargoes are considered a captured representative sample of nuclear and cytosolic factors typical of that functioning and differentiated donor cell in its differentiated organ. These could also include longer somatically mutated DNA fragments and thus presumably also L1 retrotransposons or simply DNA transposons that carry their own integrase as demonstrated by Fogarty in 2001 [64]. These donor cell proteins and nucleic acid factors (and other small molecules including cytokines, hormones, lipids, etc.) are delivered by membrane fusions into the target cell cytosol (and then to the nucleus), thus to both other somatic cells and also, potentially, to host germ cells; c.f. Cossetti et al. 2014 [16]. Post natal maternal influence secretions such as colostrum and milk carrying such vesicle/exosomal enwrapped factors, could also be delivered to progeny organisms and thus to host somatic and germ cells. These vesicle/exosome enwrapped concepts have been reviewed in detail by us and others in terms of deaminase somatic mutagenesis in the progressing cancer microenvironment [32,65]. Thus, this is not just hypothesis. It is based on a clear variety of factors and including proteins, hormones and RNA and DNA informational molecules known to be enwrapped in vesicles and exosomes in body extra-cellular fluids [16,32,65]. Indeed, there was a clear hint that these types of cargoes of small RNA molecules within endogenous retroviral vectors must have existed in the literature as early as 1981 [2].

**Figure 2:**
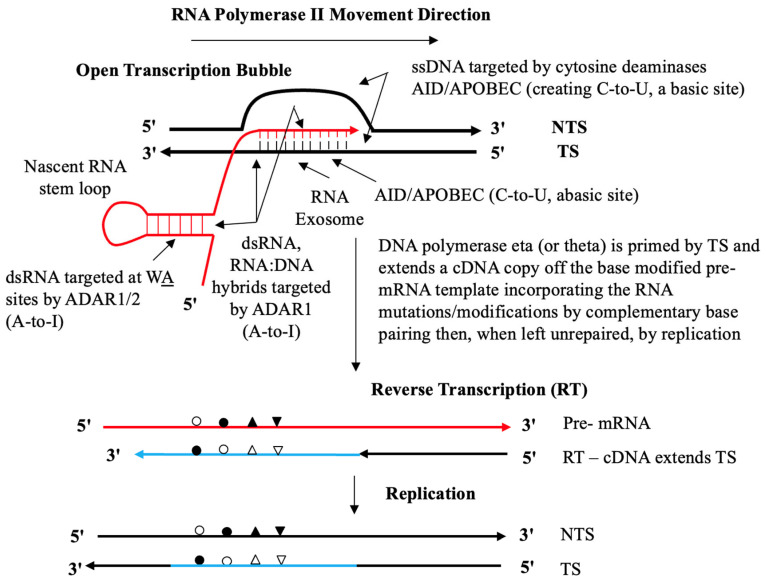
DRT Model of Somatic Mutagenesis at Stalled Transcription Bubbles. Taken from the Open Access article Figure 1a in [69,90]. More details are provided in the text. AID—activation-induced cytidine deaminase, a member of APOBEC family of cytosine deaminases, essential to initiate the SHM process at Ig loci; APOBEC family of C-to-U deaminases—apolipoprotein B mRNA-editing, catalytic polypeptide; ADAR1 and ADAR2 isoforms—adenosine deaminase acting on RNA, which execute RNA (and DNA) adenosine-to-inosine A-to-I editing at RNA:DNA hybrids [91]; TS is transcribed strand; NTS is non-transcribed strand; TSRT, target site reverse transcription [92]. The RNA Exosome action at RNA:DNA hybrids is to expose unpaired cytosines to AID deaminase on the TS as described in [93]. Alternate symbol fills are shown to symbolize RNA mutation or modification as a complementary base pairing partner in DNA. Also see and compare the prior published schematic summary showing the main elements of the reverse transcriptase (RT) mechanism for immunoglobulin (Ig) somatic hypermutation (SHM)—RT Ig-SHM—and the target-site reverse transcription (TSRT) process as a patch correction around DNA lesion sites following Luan et al. 1993 [92] and as discussed [90].

In the second phase the increased levels of RNA production make the loci susceptible to deaminase-mediated RNA and DNA somatic mutagenesis [66,67,68] and Figure 2. Thus, ‘somatic RNA mutations’, such as ADAR deaminase-mediated A-to-I RNA editing, manifest as A-to-G base changes following reverse transcription. Further, as recently analyzed [69] isomerization of U leading to pseudouridine (ψ), now behaving like “G” base pairing (manifested as A-to-C base changes following reverse transcription and target site transcribed strand DNA integration, or TSRT, Section 7). Such somatic mutations can also include AID/APOBEC deaminase-mediated C-to-U and C-to-T base changes in DNA (and RNA viz APOBEC3A) at opened Transcription Bubbles; Figure 2. These observed somatic mutation events are presumably ‘benign’, or at minimum non-lethal, as the deleterious mutations would be deleted as they emerge by purifying apoptotic events in any such donor somatic cells. While they may appear ‘benign’, subtle mutations leading to altered protein products by such coding sequence changes could enable improved functional integration into the wider functional proteome of that organ or tissue (the protein–protein interactome of the healthy cells of the somatically adapting multicellular organism). Thus, one expectation, if such ‘benign’ somatic mutations in protein coding become inherited, is that evidence should *already exist*, particularly in the modern genomic sequencing era. In support of that proposition is the observation that ‘benign’ protein coding allelic polymorphisms (SNPs) should be found in human genomic databases—and this is indeed the case for the large number of such ‘benign’ or functionally ‘neutral’ SNPs across many protein-coding gene regions curated in the Online Mendelian Inheritance in Man (OMIM) database [68]. And in a Google AI search on dbSNP (6.2.26) on the question “What is the proportion of benign SNP allelic polymorphisms in protein coding regions of the Human SNP database?” we note while “dbSNP contains millions of variants, the overwhelmingly dominant fraction represents neutral human variation (>88%)”. Accordingly, the RNA (and DNA) fragments carrying somatic mutations and their altered protein products of such events can also, in turn, be extruded as membrane-bounded vesicles allowing hypothesized potential delivery to reproductive cells and thus to germline gene repertoires in progeny organisms.

In this way, we hypothesize that a ‘Soft’ (and reversible) somatic adaptation can potentially, over time, become ‘Hard wired’ DNA Lamarckian Inheritance. Key questions remain. What is the evidence that hard-wiring lock-in steps exist, and what is the current molecular evidence consistent with ‘Hard wired’ DNA Lamarckian Inheritance? And are there identifiable key mating patterns that affect hard DNA wiring?

We now discuss key aspects of the ‘Soft’ to ‘Hard’ transition, which can historically be associated with what has been traditionally called in neo-Darwinian theory as “The Baldwin Effect.” [70].

## 3. Critique of the ‘Reversible’ Interpretative Aspects of Current Epigenetic Inheritance Demonstrations

All current experiments and human observations emphasize the ‘above the genes’ reversible aspects of epigenetic inheritance. Indeed, the consensus definition of what constitutes epigenetic transgenerational inheritance is best summed up by Ho and Buggren [71] as simply “Epigenetics, the transgenerational transfer of phenotypic characters without modification of gene sequence”. The observed transmission via the male line is always set up by mating the affected male F_1_ progeny (or later male progeny beyond the F_1_) to a normal virgin female of that inbred line. It is clear the paternally transmitted effects dissipate and wane by F_5_ or F_6_ breeding generation. This especially applies to the many recent experiments attempting to propagate the maternal stress-induced behavioral responses [26] including those experiments testing the inheritance of trained innate immunity [52]. All of the experiments brought to our attention and using small animal models employ an experimental breeding plan that specifically *avoids* brother × sister *inbreeding* or *interbreeding* among the phenotypically affected offspring of post F_1_ and later generations F_2_, F_3_, F_4_ etc. The originally modified F_1_ males and indeed in all cases, the environmentally induced signals are *only applied* on the parental generation and not applied in post F_1_ generations of males (or females) parents selected as the parents for further breeding.

We are now of the firm opinion that this breeding strategy *holds the key* to understanding how hard-wired Lamarckian inheritance occurs rapidly in the wild and in our human ancestral forebears in the evolutionary past.

In the case of past human evolution, incestual brother × sister conceptions, including among first and second cousins, are likely to have been a very common reality in small migrating or settled human groups, extended families, tribes and village communities. The clearly identifiable intra-breeding racial groups of our current era (Indian, Chinese, Japanese, European Caucasians, many Arabian societies, etc.) include distinct facial characteristics, skin color and other distinctive features such walking gait and squatting and behavioral characteristics—Steele et al. 1998 see (pp. 192–195 [5])—which may well have emerged this way over many past millennia, both prior to and then following the ‘Out of Africa’ migration expansion [72,73]. The same can also be assumed to apply in small geographically isolated groups of sub-human primates and many other animal species (both domesticated and wild) from small rodents to wild dogs to the great cats and many other species (and currently ongoing in the wild). So, the new environmental signals, whether immunological, physiological, physical or behavioral changes (now turned into stable habits and physical features), would have been sustained until further diversification took place, prompting new rounds of environmentally guided Lamarckian transgenerational rapid adaptation, and thus accelerated evolutionary diversification. This picture does indeed contradict the expectations of the slow and ponderous pace of traditional neo-Darwinian natural selection evolutionary theory of the recent past. It is akin in many ways to the whole opus of “Creative Evolution” advanced by the great French scientist, mathematician and philosopher of science Professor Henri Bergson in 1907 [74].

We make this proposal because experimental demonstrations of induced transgenerational Lamarckian acquired inheritance effects performed 50 to 100 years ago all involved the above currently deleted features viz. subsequent brother × sister inbreeding or affected progeny interbreeding to produce F_2_ and later generations, and involving either *endogenous exposure* to the new environmental signals influencing the maturation of a new generation of developing germ cells (diabetogenic, active autoimmunity) or *exogenous* signals (non-replicating and thus harmless foreign antigens deliberately delivered prior to mating in each new parental generation by the ‘*antigen-before-mating*’ breeding strategy) [1,2,54,55].

These mating strategies established stable inheritable states. We are of the opinion these constitute the *sustained new environmental changes over many generations* required by the assumptions of The Baldwin Effect to what was assumed to facilitate or mimic or simulate Lamarckian inheritance in the context of a neo-Darwinian selection paradigm [70]. These were the case whether the acquired stable state was abnormal and life threatening, such as induced diabetes [59] or induced autoimmune disease [55] or a non-life threatening altered somatic state such as the magnitude of the antibody responses of progeny to a non-replicating, harmless foreign antigen [54,75].

## 4. Hard Lamarckian Inheritance May Obligatorily Require Forms of Inbreeding Among Affected Progeny Populations Including Sustained Stimulation by Endogenous and Exogenous Signals

So, in the wild among animals and in many small and geographically isolated human populations we can assume that in, the past, brother × sister inbreeding was and is likely very common, or at the very least involved interbreeding among affected offspring (e.g., latent diabetic individuals), especially in small, closed communities including large families, clans and small tribes. Here we describe breeding experiments in laboratory animals, rabbits and rodents, that illustrate these principles.

These experiments range from straightforward simple immune antibody responses [54,75] to induced eye defects caused by autoimmune phenomena in the pregnant mother [55] to induced metabolic disorders in the parental male and female parents, such as overt spontaneously expressed or latent diabetes in later post-F_2_ generations that progressively deepened down the generations [59].

### 4.1. Low and High Serum Antibody Responses Starting with Foundation Random Bred Swiss White Mice

These are the classic well-accepted breeding experiments by Biozzi and coworkers [54,75] initiated over 60 years ago to produce stable inbred mouse lines displaying high or low antibody responses to foreign erythrocyte antigens (red blood cells, non-replicating) such as those from the sheep (SRBC). Serum antibody levels at 7 and 14 days post intravenous immunization were to select phenotypes typed Hi or Lo at high and low tails of the normal distribution as the ‘selection’ indicator. The immunization-before-mating strategy was repeated down the generations. Each generation of parents from Lo and Hi groups were interbred in groups bred by brother x sister matings or interbreeding. It took over 20 generations of inbreeding from P, to F_1_- > F_10_, F_20_ to get stable Lo and Hi antibody responder lines. A complete, easy-to-follow summary description has been published [54]. It is clear in the Lo line that an entirely new serological antigen determinant (short amino acid sequences in mouse Ig protein structure) carried on IgG (γG) antibodies viz. termed ‘allotype’ determinants as ‘3,5’ appeared and was stably inherited in the Lo line. These specificities *were not found* in the Swiss white mice foundation stock population nor in many other well-known inbred mouse lines allotyped, nor in other random outbred closed-colony mouse lines and nor were they found in many wild mice tested.

Our interpretation of these striking data 40 years ago [54] generated significant interest and controversy amongst the immunological expert community [54]. In our view this breeding program is a clear case of acquired somatically generated Ig gene mutations encoding an antibody gene structure that became inherited in the mouse germline at some point beyond F_1_. This, interestingly enough, is the same interpretation as the previous analysis of well-documented published breeding studies in closed-colony rabbits discussed and analyzed earlier as the inheritance of acquired rabbit ‘idiotypes’ by Eichmann and Kindt in 1971 [76] (‘idiotype’ refers to the amino acid structure of rabbit IgG variable region (V) genes responsible for antigen-binding and recognition [1,2]). That comparative analysis of four big rabbit breeding programs in that era 50 or more years ago formed some of the rationale for the experiments in genetically defined inbred mice with the Gorczynski laboratory, exploring evidence for inheritance of immunological allo-tolerance using male mice deeply tolerized at birth to a novel alloantigen and mated to normal immunologically naïve females of the same inbred mouse line. These studies demonstrated the transgenerational inheritance of acquired neonatal immunological tolerance (through to at least F_2_) to foreign histocompatibility antigens induced in CBA/H inbred mice foundation stock against the A/J line mouse line H-2 histocompatibility antigens as presented by heterozygous cells from CBA/H × A/J to the newborn male offspring of normal CBA/H males and female parents [77]. More defined congenic mouse lines were used in later experiments yet produced similar yet highly H-2-specific paternally transmitted responses to the F_1_ generation [78], with evidence (using two individual tolerizing alloantigens in the same male) for transmission of tolerance independently to the two alloantigens. The resulting controversies around these experiments is discussed, summarized and referenced at length in [10].

### 4.2. Chemically Induced Alloxan Diabetes in Laboratory Rats and Other Small Animals

These are the results of exemplary experiments by Goldner and Spergel [59] following some earlier similar studies in small animals of various types by Okamoto [60]. All of this literature is comprehensively reviewed by Goldner and Spergel. Their experiment was conducted in random-bred closed-colony Sprague Dawley rats. After a single shot of a sub-diabetogenic injection of Alloxan (a cytotoxic pancreatic beta-cell-targeting drug) they set up a latent diabetic phenotype in the parental generation in both male or female foundation parental (P) alloxanized rats. The intensity of the latent diabetes was measured by quantitative Glucose Tolerance Clearance curves in parents and offspring. The exposure to Alloxan only took place in the original male and female parental stock to produce the F_1_ generation and not applied to parents of later generations. The breeding beyond F_1_ involved interbreeding among affected progeny within untreated Control line F_1_–F_3_ and Alloxan-treated progeny of the sub diabetogenic dose of Alloxan-induced in parental stock. Induced glucose intolerance was transmitted by either the male or female parents to produce the F_1_.

The lines—via this interbreeding strategy—were ‘Both Male and Female Parents Alloxanized’, ‘Male Parent only Alloxanized’, ‘Female Parent only Alloxanized’, and already a clear sex difference in control and test progeny was evident; females always had slower glucose disappearance rates than males. It was obvious that glucose disappearance rates deteriorated through succeeding generations. By the F_3_ the deterioration was far worse quantitatively than the F_2_, which itself was lower than the F_1_ compared to the Control F_1_–F_3_ population that showed no shift down in glucose clearance rate from original Control rats.

So, the latent diabetogenic state deepened down the breeding generations. Goldner and Spergel review the other studies of that era that were inspired by original experiments of Okamoto [60]. Okamoto used rats, rabbits, and guinea pigs with high diabetogenic doses of producing overt high blood glucose levels F_1_–F_7_ and exposing each new parental generation to alloxan treatment. The emergence of overt ‘spontaneous diabetes (high blood glucose)’ then began to appear without alloxan treatment of parents by F_6_, F_7_, and transmitted further via the male line. Goldner and Spergel [59] provide a good summary of the main Okamoto findings: “for the first time, it was shown that it was not only the diabetic or possibly diabetogenic milieu of the mother, nor some unknown milk factor that was responsible for the transmission of diabetes. When spontaneous diabetes did develop in Okamoto’s animals, almost all the affected generation had diabetes, though in varying degrees of severity”.

In quite a large study as judged by progeny numbers, one of us attempted a limited reproduction of this paternal transmission of chemically induced diabetes in inbred CBA/H mice [79]. The strategy used the β-cell targeting drug streptozocin (STZ) and merged the Okamoto and Goldner–Spergel approaches ensuring good breeding and progeny numbers. A single high dose of STZ or repeated low doses of STZ were used in the 5 weeks in the males prior to mating with normal females (at 10 weeks age). The main observation in the F_1_ of the breeding fathers given a single high dose STZ (overt high blood glucose or hyperglycemia) or multiple sub-diabetogenic doses of STZ was a significant shift to an elevated mean weaning body weight (a general developmental metabolic disorder). However, only a single overt spontaneous diabetic (high blood glucose) progeny mouse was observed, and that was from a single high dose STZ diabetic father crossed with a normal female.

### 4.3. Inheritance of Autoimmunity-Induced Eye Defects Caused by Anti-Eye Lens Immune Responses in Progeny of Immunized Pregnant Mothers with Eye Lens Self-Antigens

Guyer and Smith in 1918–1924 [80,81,82] demonstrated that administration of pregnant rabbits with fowl-derived antibodies directed against rabbit ocular lens antigens—normally sequestered from immune surveillance as essential self-antigens located within immunologically privileged sites—resulted in transplacental transfer of these antibodies and the subsequent birth of offspring exhibiting severe ocular malformations. In other experiments they injected the pregnant rabbits directly with eye lens homogenates, thus immunizing the mother against her own eye-lens antigens. In both cases such mothers passed the antibodies across the placenta, and the developing babies became affected, and many were born with severe eye defects. They showed that these induced eye defects were passed on to future generations of progeny rabbits via the male or female lines without any further immunization or antiserum treatment of subsequent parental rabbits with eye lens antibodies [80,81,82]. A full description of these studies can be found in [55].

Thus, the experiments demonstrated progressive loss of eyes and severe eye abnormalities in successive generations of brother × sister or interbreeding of defective eye offspring with the worsening condition transmitted via defective eyed male or female parents in the F_1_ down to as far as F_9_. So, an escalating and a very severe autoimmune condition presumably involving both antibody-producing specific B lymphocytes and presumably also T helper (Th) and regulator lymphocytes (Tregs) was stably transmitted, implying genetic modifications at the hard-wired germline DNA level. We speculate that some of the changes among germline genes of the immune system involved intense somatic selection and production of high binding affinity Ig V regions specific for self-rabbit eye-lens proteins. That is, the genetic changes likely involved major updates and additions to the rabbit germline V element arrays. These arrays are inherited as long tandem-like arrayed haplotypes (~1 Mb or greater, see Section 7, Section 8 and Section 9).

The key difference from all contemporary epigenetic transgenerational experiments via the male line is there was no attempt to mate only to a normal untreated line female (i.e., normal females). Clearly the strategy in this escalating condition is to allow interbreeding of overtly affected or latently affected progeny in generations post F_1_. We interpret this as implying that the key to stable Hard epigenetic-induced inheritance thus actually requires brother × sister or interbreeding in a closed affected lineage group.

## 5. Lessons for Future Lamarckian Breeding Programs

The lessons for future experiments for affecting, and then detecting, ‘Soft’ to ‘Hard’ Lamarckian epigenetically-induced inheritance needs to be acknowledged. Many of the technical tools, not available just 10 years ago, now allow large, long-read haplotype genomic sequencing in the Mb range, as have been developed by the Watson and Collins groups [48]. This allows phased linkage analysis at the nucleotide level of the multiple tandem arrays of key similar yet specifically different immune-antigen-binding IgV- and TCRV-specific germline elements. In theory, this approach could also be applied to the similar large haplotypic arrays of olfactory volatile ligand-binding G protein receptor genes that could be applied as a genetic screening approach to the progeny in the Pavlovian conditioning experiments of Dias and Ressler [19].

## 6. Reflection on Other Historical Acquired Inheritance Experiments and Observations Involving Paternal Influence, the Sire Effect and Maternal Influence

The fact that paternal serum factors from maternal-stress-exposed males can transfer stress-induced paternal metabolic phenotypes to progeny [24] suggests vesicles/exosomes in wider blood and lymphatic circulation carrying cargoes of miRNA, lncRNA and protein or lipid regulatory molecules. We suggest these circulating factors are akin to those factors in milk affecting cross-fostered offspring of apparently normal mothers mated to neonatally tolerized males made hyporesponsive to CBA × A histocompatibility antigens identified by Gorczynski et al. in 1983 [83]. This is also consistent with the milk factors in later cross-fostering observations by Gorczynski and associates in Pavlovian conditioning breeding experiments [84] as well as the Sire Effect of Sobey and Connelly in the deliberate Myxomatosis Rabbit plague control in 1940s and 50s in regional Australia, reviewed in Lindley 2010 see (pp. 26–27 [7]).

Gorczynski conducted Ader and Cohen [85]. Pavlovian conditioning experiments in mice, in which cyclophosphamide-induced immune suppression was linked with saccharin as the conditioning regime in the drinking water. They showed both a saccharin-mediated conditioned recall of immune (as initially described by Ader and Cohen) but also the transmission to offspring for several generations of that conditioned immunosuppression. The conditioned immune suppression was localized to a transmissible entity by a maternal cross-fostering design to the characteristics of the nursing foster mothers [84]. In later experiments these causal effects were localized to regulation by factors in the colostrum/fetal-placental unit modified by the conditioning phenomena [86]. Indeed, this conclusion was the same as the maternal immune factors transferred in the acquired sire effects reported earlier [83]. We now infer these were key unrecognized factors at the time in the earlier controversial Gorczynski–Steele experiments published in 1980, 1981 [77,78]. We would now hypothesize that such factors in circulation and milk could have affected the phenotype of the hypo-responsiveness of cytotoxic T lymphocytes in the F_1_ (and later) generation male from CBA/H mice and other defined congenic strains, made neonatally tolerant to foreign H-2 transplantation antigens [77,78].

So, in an irony of history returned now as a real ‘fact of life’, these are the vesicles with enveloped nucleic acids that align conceptually with the ‘Pangenes’ or ‘Gemmules’ envisaged by Charles Darwin’s highly original theory of Pangenesis [5,11,12,13] to explain the acquired inheritance phenomena he observed in plant-grafting experiments. These types of plant-grafting genetic effects are discussed at some length by Yongsheng Liu in Steele et al. 2019 [10]. With respect to the penetration of the Weismann Barrier, we need also to cite the important experiments of the Spadafora group on soma to germline transmission over many years, which clearly shows the consistency of current observations consistent with Darwin’s theory of Pangenesis Spadafora; Cossetti et al. [15,16].

Thus, nucleic acid regulatory informational molecules can facilitate putative cell-to-cell transfer of genetic information and, being enwrapped in vesicles, could putatively deliver new somatic genetic information to other somatic cells and to the germline. At this juncture it is relevant to cite the prior work of a young MD student in Ireland, Wilfred Chen, who, to our knowledge, was the first to formally discuss such lateral and vertical genetic cell to cell spreading processes. On the basis of those studies he postulated the Hypothesis of Genetic Exchange in his 1968 MD thesis titled “Intercellular exchange of genetic material in the control of tissue growth and Differentiation”, National University Ireland (NUI), which states “there exists a system of exchange of intercellular factors, including genetic material (DNA/RNA) important for the homeostatic control of growth and differentiation. Any disturbances of the exchanging system, or the messages themselves, result in abnormal growth even cancer”. This was later published in the West Indian Medical Journal [87].

However, an unanswered question remains: How then do these newly arriving RNA informational molecules become locked into germline DNA, thus resulting in ‘Hard Lamarckian Inheritance’? We believe our recent work on ‘Deaminase-Driven Reverse Transcriptase Mutagenesis and Target Site Integration at Ig and Non-Ig Loci’ across the cancer genome likely provides some understanding of a general mechanism at the molecular level for an Epigenetic–Genetic Coupling schema as outlined in Section 7.

## 7. A General Mechanism for Donor Nucleic Acid Recognition and Integration of Target DNA Base Sequences

What is a plausible mechanism for generating donor nucleic acid sequences, especially long and short RNAs, that allows both recognition of a target sequence and then delivery of the donor sequence such that it *replaces* the resident genomic sequence, and whether that sequence resides in another somatic cell or a germ cell? In this way the resident sequence can be cleanly removed, and the donor sequence integrated exactly in its place in genomic DNA and thus be replicated to progeny daughter cells and organisms. This is a crucial step, even if potentially sloppy and error prone—and it is not often openly addressed directly in discussions on mechanisms of evolution. Yet this is exactly what must happen in any process expected to execute Lamarckian acquired inheritance adaptations. We recognized this early on (e.g., reviewed in [1,2,3]) as a crucial step for clean integrations into the germline DNA of incoming pre-mRNAs encoding somatically rearranged and mutated immunoglobulin V region genes via the then-predicted soma-to-germline transmission process [1,2].

### 7.1. What Is a Likely Hypothesized Recognition Step in the Stochastic Search for the Target DNA Sequence in Epigenetically Opened and Accessible Chromatin?

**Figure 3:**
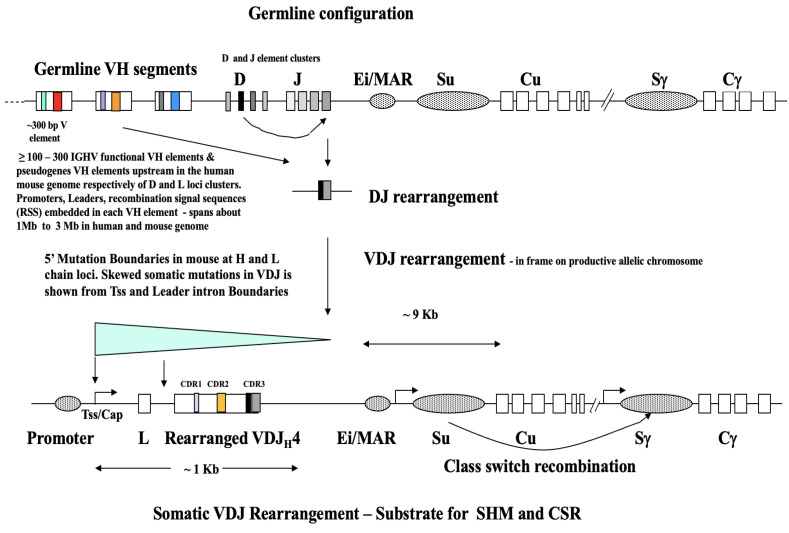
The Somatic Rearrangement of a Typical Immunoglobulin (Ig) Heavy (H) Chain encoding Variable (V) gene in the IGHV locus of the Human or Mouse Genome. This is an illustrative schematic figure showing the somatic rearrangement of a given, ‘stochastic or randomly selected’ germline encoded IGHV heavy chain VH element from the upstream V element array to produce an in-frame productive and transcriptionally active ‘V– > VDJ’ rearrangement at the IGHV heavy chain locus. Light-chain encoding arrays have a similar structural layout, yet no D elements, only J elements. The generic symbol V[D]J denotes a somatic rearrangement to cover DNA rearrangements of both heavy- and light-chain loci. The other allelic chromosome is inactive (termed ‘Allelic Exclusion’). Note that in a maturing B lymphocyte clonal lineage the D– > J rearrangement precedes the V– > DJ step. The figure at the top shows the germline configuration of an array of unrearranged V elements on the left, to the D and J (diversity, joining) element clusters which on the activated allelic chromosome creates the substrate for somatic hypermutation in that clone by what is known as V– > VDJ rearrangement. The V elements are structured and highly non-random, consisting within a V family of highly conserved framework regions (FW) and highly variable short complementarity-determining regions (CDR) that encode the amino acids that make contacts with antigenic epitopes. The CDRs are indicated as short colored bars within the body of the V element and the FW left unfiled. So, in a typical putative functional V region the order 5′ to 3′ over about 100 triplets or amino acids is FW1,CDR1, FW2, CDR2 then FW3 which leads into the 5′ border of what will become CDR3b when fully rearranged, the DJ regions are also highly variable, making up CDR3 in a fully rearranged V[D]J region. The rearranged V elements, normally silent and unexpressed in the germline configuration, is now activated and produces pre-mRNAs (which on processing can be translated into a mature heavy (H) chain protein for heterodimer association with a similar light (L) protein from a similarly rearranged light (K, λ) chain locus (which, as indicated, only has J elements). The ‘Somatic V[D]J Rearrangement’ is the substrate for somatic hypermutation (SHM) and immunoglobulin class-switch rearrangements (CSR), usually in post immunization (antigen-drive) Germinal Center primary lymphoid structures, and then proceeding in a programmed fashion though IgM (constant region Cu chains), to IgG subclasses (Cγ chains), or IgA (Cα chains) or IgE (Cε chains) antibody molecules. After rearrangement, all intervening bits of contiguous DNA sequence on the productive allele are deleted and lost from the locus. Thus, if a human IGHV rearrangement is to the VH4-39*01 element (c.f. Figure 1 in Watson et al. [33]) all intervening DNA down to the rearranged DJ is lost, as is the DNA sequence upstream of the DJ fused region, any downstream unrearranged J regions in the J-C intronic region will remain in place. The illustrative schematic figure is not drawn to scale and is adapted and modified from previous papers [94,113,114]. While this is an example at the human or mouse heavy-chain locus, the human and mouse IGHV haplotypic loci are similar in principle, as are the loci for immunoglobulin light chains (IGKV, IGLV) and in T cell receptor V loci (TCRV) for gamma (γ) and delta (δ) chains and other related TCR chains.

This is a crucial first step in the integration process (discussed further in Section 7.2). The work initiated by Mattick [27,28] and then in key papers on ‘Triplex’ sequence recognition not requiring conventional A•T and G•C hydrogen bonded base pairing is very important [29,30,31] and reviewed later also in Li et al. [88]. Thus, long non-coding RNAs (>200 nt) which target flanking regulatory regions of protein-coding genes are now a regulatory epigenetic factor [27,28]. LncRNAs specifically target DNA sequences usually via RNA–DNA triple-helix interactions which allow weaker Hoogsteen hydrogen bonding yet biologically significant in long sequence recognition of dsDNA helices. These Hoogsteen base pairings, much weaker than A•T and G•C Watson–Crick base pairing, can be varied, in both parallel and anti-parallel configurations with the RNA sequence aligned in the major groove of the DNA duplex [88]. Thus, multiple points of H-bonded duplex DNA over far longer significant sequence lengths (such as long protein-coding genes bounded by Enhancer or Promoter regions) allows gene-specific recognition via RNA–DNA triple-helix interaction. Indeed, such a recognition step allows targeted delivery of chromatin modifications which can result in either active transcription (activation via acetyltransferase-associated complexes) or gene silencing (chromatin compaction via methyltransferase-associated complexes). These lncRNA epigenetic regulators are ubiquitous in secreted extracellular vesicles and exosomes tumor cell microenvironments [89]. We propose this step as a key first step in the stochastic search for and then sequence recognition (in dsDNA), thus guiding the subsequent target site reverse transcriptase (TSRT) integration step proposed below for other transcribed pre-mRNAs which now act as donor coding sequences at the epigenetically opened and actively transcribed locus, e.g., the array of unrearranged V element sequences at IGHV (Figure 3).

This then is an essential part of the hypothesized targeted epigenetic opening step at key target genomic loci. This is associated with RNA transcription upregulation and pre-mRNAs synthesized at a higher rate, thus making that transcribed locus *potentially vulnerable* to Deaminase-mediated Reverse Transcription (DRT) mutagenesis. This is discussed in both in the reverse transcriptase mechanism of somatic hypermutation of rearranged immunoglobulin genes [90] and at non-immunoglobulin loci across progressing cancer genomes [69] and Figure 2.

### 7.2. A Proposed General RNA-Templated Reverse Transcriptase Mechanism for Targeted Integration of Donor Sequence by Homologous Recombination into Somatic Cell or Germ Cell Sequence Loci

This step is ideally suited for the plausible *gene replacement* by donor RNA sequences at highly similar repetitive loci involved in general ‘environmental signal stimulation’, encoding such receptors as Immunoglobulin antigen binding V regions, T Cell Receptor V regions, MHC Class I and II peptide antigen binding receptors that present processed peptide antigens to T cells, or Olfactory G Protein Receptor arrays for volatile environmental odorant ligands. Some of the specific principles are discussed in depth [94].

The recent work on deaminase-driven reverse transcriptase mutagenesis signatures (DRT) in thousands of cancer genomes, and thus putative target site integration at non-Ig loci across the somatically evolving cancer genome, is now important to consider [69]. The key enzyme executing TSRT (Figure 2) is the ubiquitous DNA repair polymerase DNA polymerase eta (η). As well as being a DNA-dependent DNA-repair polymerase, this is also a very efficient RNA-dependent DNA-repair polymerase or ‘reverse transcriptase’ [95,96,97]. DNA polymerase η has now been clearly demonstrated by Chakraborty et al. 2023 [98] to execute RNA templated error-free DNA repair of double-strands breaks (DSBs) in the human transcribed genome and thus restore the missing sequence at the DSB site via a transcription-coupled nonhomologous end-joining (TC-NHEJ) pathway. In theory this should also easily be applied to and demonstrated for both allelic (AHR) and non-allelic (NAHR) homologous recombination among highly similar target sequence sites—such as the long germline IgV element haplotype arrays (≥1 Mb, e.g., IGHV) as illustrated in Figure 1 of Steele and Lloyd [94], or long (4 Mb) germline MHC Class I and Class II and ancestral haplotypic loci, or indeed long Olfactory G protein gene receptor signaling arrays. The Chakraborty et al. [98] demonstration in human cells (stable human embryonic kidney cell lines, HEK293 cells) is a major advance both technically and conceptually, as it confirms a key prediction of the reverse transcriptase mechanism of Ig SHM [90,95,99] and thus the generalized nature of the TSRT process (Figure 2).

Such a molecular outcome, delivered by donor RNA or pre-mRNA-loaded vesicles/exosomes, may by necessity be part of the hypothesized inbreeding/interbreeding genetic lock-in step flagged in the discussion above. This proposed outline is only a guide, and the detailed cellular and molecular steps remain to be discovered by future experiments. However, it is plausible, as it fits all extant published evidence. We should stress all these suggestions arise from our work on understanding the reverse transcriptase mechanism of Ig somatic hypermutation (SHM) in somatically rearranged Ig variable region genes (see Figure 3) and now extended to non-Ig loci of protein coding genes and especially in the thousands of now fully sequenced progressing cancer genomes [69] curated at the Sanger COSMIC database [100,101,102].

The molecular steps in this genetic locking-in integration process are schematically summarized in Figure 2.

The unpaired single stranded (ss) DNA sites in the open transcription bubble are targeted by the AID/APOBEC cytosine deaminases creating C-to-U and Abasic lesion sites. The black strands represent DNA. The red strands represent RNA. And cDNA is represented by blue strands. RNA mutations (G-to-A, G-to-C, G-to-U) can emerge via the RNA Polymerase II complex transcribing *without a block or stalling* across these AID/APOBEC cytosine deamination lesion sites [103] and these are indicated by open circles. The RNA exosome allows access to unpaired cytosines on the TS in the RNA:DNA hybrid [93]; RNA mutations via transcription-coupled ADAR1 deamination of adenine to inosine (A-to-I) in the nascent dsRNA [104] or on both nucleic moieties of the annealed RNA:DNA hybrid (9–11 nt) indicated by closed circle [91,105]. Other subsidiary non-deaminase-driven RNA modifications could include endogenous uracil isomerization to pseudouridine (ψ) to give a U-to-G miscoding substitution indicated as closed triangles (see references in Steele and Lindley [69]); or non-deaminase-driven RNA miscoding mutations (G-to-U) following reactive oxygen species (ROS) generation of 8oxoG in nascent RNA or the annealed RNA:DNA hybrids [106], indicated by inverted closed triangles. The last TSRT step is effectively a potential ‘error prone’ DNA repair process itself akin to a patch nucleotide excision repair (NER) on the TS allowing replication of the helix in that damaged genomic region, discussed at length in [107].

In this way, target-site reverse transcription (TSRT) executes a clean replacement integration of the RNA copy now as integrated genomic DNA at that locus site. The deaminase-driven reverse transcriptase (DRT) mutagenesis process [69] involving RNA templated DNA Polymerase-η (eta) reverse transcription we predict is a general process. This drives what we now consider to be a universal homologous integration phenomenon whether at allelic or non-allelic sites *provided sufficient sequence similarity* (say ≥75–80%) between donor and target DNA sequence exists. This allows an understanding of how a hypothesized extreme putative transgenerational “churn-like” might occur. The extremely diverse sequence structure among germline VH element arrays may arise this way [108,109,110,111]. This diversity is also seen in the inferred haplotype data of Kidd et al. [46] and Watson and associates [33,36] within the confined I Mb region of the long haplotype at the IGHV-IGHD-IGHJ-IGH Constant Region locus at band 14q32 on human Chromosome 14. We discuss this as a hypothesized *expected outcome* of DRT/TSRT integration events further below.

## 8. Basic Immunology—An Approach to Acquired Inheritance Breeding Programs to Secure Hard Lamarckian DNA Sequence Evidence

One big goal of our paper is to provide an experimental road map of conceptual options to drive future experimentation in acquired inheritance breeding programs. One possible vehicle would be to use genetically defined inbred strains of mice; however, we believe securing donor blood from healthy human volunteers from members of large extended three-generational families is the preferred option to understand how Lamarckian Inheritance continues to rapidly shape human evolution (c.f. the long haplotype strategy outlined in [94]).

Why explain in some detail the basic immunology of the somatically DNA rearranging antigen binding receptor genes (Figure 3)? We do this because in our long experience there is little understanding of somatic rearrangement at immune receptor arrays, and that SHM is only executed on rearranged V[D]J genes. The V elements themselves in the germline configuration are not expressed, thus are not acted on directly by antigen-mediated binding of antibody or T cell receptor protein heterodimers. This means that Ig and TCR V element receptor arrays are *not direct targets of conventional Darwinian antigen binding selection*. Such selection only occurs on the *somatically mature* B and T cells with the protein products of V[D]J productive rearrangements viz. heterodimer antigen binding sites displayed on the B or T cell surface. The two other major genomic tandem arrays of ligand binding receptors which do not somatically rearrange are the G signaling protein encoding olfactory binding gene arrays and the foreign protein antigen presenting molecules of the Major Histocompatibility (MHC) loci arrays—encoding Class I and Class II molecules, presenting short processed ~20 amino acid antigen peptides to cytotoxic T cells (Tc) and T helper and T regulator (Th, Treg) respectively. For B lymphocytes that produce antibody heterodimer (heavy plus light chain) antigen-binding sites the key point is that all subsequent antigen-binding events, cell selection and stimulation and B lymphocyte somatic hypermutation (mainly in Germinal Centers) *only take place on the V[D]J somatic rearrangement* that defines the initial antigen specificity of that cell, and subsequent expanded clone. This was all discussed at length 28 years ago when we outlined how the signature of antigen-driven SHM and somatic selection appears in the germline DNA [4,5]. As far as we are aware the only other rearranging genomic array loci outside the immune system is in the brain. The genes encoding receptor molecules in receipt of ‘environmental’ signals in the broadest sense are those encoding the synaptic receptors of the brain and CNS which are driven by transposition and deletional rearrangement (as Figure 3) by L1 element retro-transposition [112].

We considered this short background essential in understanding not only the somatic hypermutation process per se, but also the prior and ongoing work of the Collins [44,45,46,47,48,49] and Watson groups [33,34,35,36,37,38,39,40,41,42,43] on the reliable identification of each germline and expressed V[D]J V element in the complex human and mouse genome so that researchers can identify a *genuine* new somatically mutated VDJ during an immune response and not the re-discovery of an already existing germline V element. As discussed [94], the generally used IMGT database [115] has always been employed with an ‘error tolerance’ awareness by the ‘adaptive immune receptor repertoire’ (AIRR) research community (see the responses to a 2015 questionnaire sent out in [94]). Correct identification would seem to be a basic requirement in all ‘adaptive immune receptor repertoire’ (AIRR) analyses underway in many investigations. The Open Germline Receptor database (OGRDB) and incorporating the expressed functional VDJ database has now become possible [42] because of the prior and ongoing work of the Watson and Collins groups. For many years the IMGT database for Ig and TCR germline V genes has been and is still used, but because of the many confusing entries which could simply be derivatives of somatically mutated V variants in that individual, this is now being superseded by OGRDB [42].

## 9. Long Read DNA Sequencing and Analysis of Immunoglobulin (Ig) and T Cell Receptor (TCR) Large Germline Arrays in ≥1 Mb Range

In contrast then to non-rearranging receptor gene arrays, the immunological situation is far more complex and potentially confusing for molecular and cell biologists that work outside the field. Thus, we close by discussing how some molecular immunologists are now analyzing to a nucleotide level of accuracy, the germline Variable (V) and linked Constant (C) genes and the associated somatic rearrangement diversity (D) and joining genes located in long phased haplotypes of B lymphocyte producing antibodies and T Lymphocyte receptor gene families. This work by several groups has revealed how plastic and complex these antigen specific V element germline arrays really are. The first fully sequenced human IGHV locus was by the Honjo group in 1998 [108]. This was built from accurate sequencing at the time of three long contigs (from three different human beings) spanning 1 Mb.

The pictural summary in “Figure 1” in [108] is very revealing in itself. It took an additional 15 years for another approximately full-length 1 Mb IGHV locus as a fully contiguous haplotype to be sequenced in a single individual to nucleotide resolution [33]. The IGHV haplotype is typified as a clearly jumbled array of about 44 functional VH family elements (including transcribed and open reading frames), 79 non-functional point mutation pseudo genes or truncated VH fragments (5′ only, 3′ only or both 5′ and 3′ truncated). Each functional VH is centrally embedded within a segment surrounded by flanking sequence (a segment anywhere from 6 to 20 kb in median length). Segmental duplication and transposition to elsewhere in the IGHV locus defines their location within the locus. There are about 7–9 VH families, each with characteristic sequence similarity by oligo nucleotide hybridization and PCR primer sequence that are defined (VH1, VH2, VH3, VH4, etc.). However as mentioned their order across the 1 Mb haplotype is ‘jumbled’ or scrambled (unlike mouse inbred IGHV families, which tend to be more numerically ordered, and expansions and contractions in V repertoire size and arrangement more readily explained by meiotic unequal crossing over).

It is for this reason we are of the opinion that such a long 1 Mb jumbled array of different yet similar sequences like the human IGHV has been subject to transgenerational soma-to-germline “churn” and impact events via incoming (at each parent-to-offspring generation) of somatically mutated VH portion of the pre-mRNAs (as part of a VDJ pre-mRNA). We have previously published the evidence on the intricate antigen-driven somatic selection signature of somatic hypermutation now embedded in IGV germline loci [4,5]. It is also the reason we previously advocated the systematic investigation of freshly collected genomic IGHV DNA from large consenting generational human families to prove this point [94]. This is why we now focus on the pioneering and ongoing work of Andrew Collins and coworkers and the work of Corey Watson and coworkers. Their systematic approach to the germline and expressed IGV repertoires is slowly revolutionizing our understanding of the genetics and diversity of these quite remarkable recognition elements crucial to the adaptive immune response to foreign pathogens. The technical lessons and expertise developed since 2013 by the Watson group have a direct bearing on how future similar yet large germline receptor G protein loci can be approached and analyzed, especially the large tandem haplotypic germ-line arrays of olfactory odorant ligand binding receptors—of direct applicability to analyzing the potential germline inheritance of somatic mutations in these genes. This is especially relevant to the Pavlovian conditioning ‘epigenetic’ inheritance studies of Dias and Ressler [19,20].

What can we theoretically expect from such systematic investigations, especially of IGV? The big one we believe will be validation of the major conclusion by the group of Honghua Li and associates over 20 years ago [109] from a systematic nested PCR analysis of the major human VH4 and VH3 germline family of elements in individual spermatozoa. They came to the extraordinary conclusion ‘‘It is believed that no chromosomes contain the same set of VH gene segments…’’. Later work by Kidd et al. [46] also imply the same conclusion (and personal communication A Collins ca. 2015). By extension, this implies—in contrast to what we expect to be the closed finite number of 10,000–100,000 4 Mb long MHC ancestral haplotypes (i.e., many repeat isolations within members of a defined intra-breeding racial group, e.g., among Caucasians) in the entire extant human population—that for IGHV (say) the unique haplotype number can be expected to be in the range of 14—16 billion in a human population of 7–8 billion [94]. These extraordinary projections need to be tested, confirmed or refuted or modified by future investigations.

We firmly believe that systematic investigation of IGV germline long inherited haplotypes will reveal the signature of real time “Soma to Germline Lamarckian churn” down the generations—as individual human groups and their extended families adapt to new challenging pathogen environments. This we expect to apply also to all the other diverse environmental signals impacting human beings at present and in the future: metabolic, odorant, behavioral and mental responses. We also anticipate some novel surprises from the informative large three generation human families using the Watson group long read single molecule sequencing protocols.

## 10. Concluding Remarks

In summary, we predict new and highly significant Lamarckian genetic inheritance phenomena will be revealed in the future by controlled experiments and observations using targeted long haplotype sequencing. These studies will likely come from human families where Epstein–Barr Virus (EBV)-transformed Lymphoblastoid Cell Lines (LCL) DNA are currently available at the Coreil depository or can be secured fresh from donor volunteers. Current work shows that peripheral blood mononuclear cells (PBMC) can serve as a source of individual genomic DNA preparation of IGHV from three generation family members containing *no somatic DNA rearrangement contamination* [34,36]. Thus, a clear new acquired inheritance allelic gene discovery strategy based on accurate long-read single-molecule sequencing *and* informative three-generation pedigrees in humans is now technically feasible.

## Data Availability

All data and observations analyzed in this paper are from previously published papers in the peer reviewed journal literature.

## Supplementary Materials

The following are available online at https://www.mdpi.com/article/10.3390/ijms27042003/s1.

## Abbreviations

**Glossary Box of Complex Terms and Key Concepts**
There are complex terms in this paper which on review demand further clarification. See Section 5, Section 6 and Section 7 for complete explanations.
**SHM and Mechanism**—SHM stands for Somatic Hypermutation in Rearranged Immunoglobulin Variable (V) Region genes see Figure 3. Until a few years ago the dominant mechanism of SHM was termed the “AID Cyto-sine-to-Uracil DNA Deamination Model.” However, the evidence has accumulated significantly in recent years in support of the reverse transcriptase mechanism (**RT Ig SHM**).
**Pre-mRNA, TSRT, SHM**—Pre- mRNA contains unprocessed intronic sequences. Under the target site reverse tran-scriptase (**TSRT**) mechanism of **SHM** this is the RNA template on which DNA Polymerase eta (η) is hypothesized to extend the nicked Transcribed Strand (**TS**) for integration and thus replace the TS in that region of double stranded DNA. Note the pre-mRNA can carry somatic mutations.
**Triplex Recognition and Hoogsteen base pairings**—A three layered ’Triplex’ DNA and RNA complex involving double stranded DNA helix layered with single stranded RNA where the recognition of sequence aligns via Hoogsteen non-H bond pairing (e.g. A pairs with A, G pairs with G , C pairs with C, U pairs with T). Hoogsteen base pairings, is much weaker than A•T and G•C Watson-Crick base pairing but can extend over large stretches of dsDNA helix.
