# Supplementary material for: Epigenetic–Genetic Coupling and Understanding the Molecular and Cellular Basis of Lamarckian Inheritance
Source: Int J Mol Sci. 2026 Feb 20;27(4):2003. doi: 10.3390/ijms27042003 (PMC12941027; doi:10.3390/ijms27042003)
Supplement: Supplementary file 1 [file ijms-27-02003-s001.zip › ijms-4123903-supplementary.pdf]

Supplementary Information to go with

Epigenetic-Genetic Coupling and Understanding the Molecular and Cellular Basis of Lamarckian Inheritance By Robyn A Lindley, Reginald M Gorczynski and Edward J Steele

Glossary Box of Complex Terms and Key Concepts

There are complex terms in this paper which on review demand further clarification

SHM and Mechanism - SHM stands for Somatic Hypermutation in Rearranged Immunoglobulin Variable (V) Region genes see Fig 3. Until a few years ago the dominant mechanism of SHM was termed the "AID Cytosine-to-Uracil DNA Deamination Model." However, the evidence has accumulated significantly in recent years in support of the reverse transcriptase mechanism (**RT Ig SHM**) see ref [90] for update. RT Ig SHM is a further expansion of the DNA Deamination Model as it incorporates that mutation process. A key development at that time was the discovery in the human lymphoblastoid cell line Ramos that the DNA repair accessory factor Ubiquitinated PCNA was required for a full balanced spectrum of somatic mutation at A/T and G/C base pairs. Please see ref 36 in our paper [90] viz. Lerner et al 2022 Expression of Constitutive Fusion of Ubiquitin to PCNA Restores the Level of Immunoglobulin A/T Mutations During Somatic Hypermutation in the Ramos Cell Line, Front. Immunol. 13 (2022), 871766. We thus speculated at that time that... "with A/T mutagenesis fully restored by ensuring optimal PCNA functional expression, rapid progress by the field can now be expected in analysing by in vitro tissue culture the full Ig SHM mutation spectrum at A/T and G/C base pairs, including a role for the RT property of DNA Polymerase η." To our genuine surprise that 'Proof' establishing the ability of DNA Polymerase eta (η) to execute target site reverse transcription (**TSRT**) had already appeared in the biochemistry field of double strand break DNA repair (DSB repair) in the human cell line HEK293 see ref [98] in the present paper.

Pre-mRNA, TSRT, **SHM** – Pre- mRNA contains unprocessed intronic sequences. Under the target site reverse transcriptase (**TSRT**) mechanism of **SHM** (Fig 2) this is the RNA template on which DNA Polymerase eta (η) is hypothesised to extend the nicked Transcribed Strand (**TS**) for integration and thus replace the TS in that region of double stranded DNA (Fig 2 and [90]). Note the pre-mRNA can carry somatic mutations as indicated (Fig 2 and [90]). As indicated, this has been shown in principle in the field biochemistry and DNA repair [98], and we await that demonstration within the field of SHM in molecular immunology.

Triplex Recognition and Hoogsteen base pairings – A three layered 'Triplex' DNA and RNA complex involving double stranded DNA helix layered with single stranded RNA where the recognition of sequence aligns via Hoogsteen non-H bond pairing (eg A pairs with A, G pairs with G etc). Hoogsteen base pairings, is much weaker than A•T and G•C Watson-Crick base pairing but can extend over large stretches of dsDNA helix.
